# Aberrant Expression of Non-Coding RNAs in Pediatric T Acute Lymphoblastic Leukemia and Their Potential Application as Biomarkers

**DOI:** 10.3390/genes16040420

**Published:** 2025-03-31

**Authors:** Neila Luciano, Luigi Coppola, Giuliana Salvatore, Pasquale Primo, Rosanna Parasole, Peppino Mirabelli, Francesca Maria Orlandella

**Affiliations:** 1 Department of Medical, Movement and Wellbeing Sciences, University of Naples Parthenope, 80133 Naples, Italy; neilaluciano14@gmail.com (N.L.); giuliana.salvatore@uniparthenope.it (G.S.); 2 CEINGE-Biotecnologie Avanzate “Franco Salvatore”, 80131 Naples, Italy; 3 UOS Research Laboratories and Biobank, AORN Santobono-Pausilipon, 80122 Naples, Italy; l.coppola@santobonopausilipon.it (L.C.); p.primo@santobonopausilipon.it (P.P.); p.mirabelli@santobonopausilipon.it (P.M.); 4 UOC Clinical and Translational Research, AORN Santobono-Pausilipon, 80122 Naples, Italy; r.parasole@santobonopausilipon.it

**Keywords:** lncRNA, miRNA, pediatric T-ALL, biomarkers, therapeutic targets

## Abstract

Less than 5% of the DNA sequence encodes for proteins, and the remainder encodes for non-coding RNAs (ncRNAs). Among the members of the ncRNA family, microRNAs (miRNAs) and long non-coding RNAs (lncRNAs) play a pivotal role in the insurgence and progression of several cancers, including leukemia. Thought to have different molecular mechanisms, both miRNAs and lncRNAs act as epigenetic factors modulating gene expression and influencing hematopoietic differentiation, proliferation and immune system function. Here, we discuss the most recent findings on the main molecular mechanisms by which miRNAs and lncRNAs are involved in the pathogenesis and progression of pediatric T acute lymphoblastic leukemia (T-ALL), pointing out their potential utility as therapeutic targets and as biomarkers for early diagnosis, risk stratification and prognosis. miRNAs are involved in the pathogenesis of T-ALL, acting both as tumor suppressors and as oncomiRs. By contrast, to the best of our knowledge, the literature highlights lncRNAs as acting only as oncogenes in this type of cancer by inhibiting apoptosis and promoting cell cycle and drug resistance. Additionally, here, we discuss how these molecules could be detected in the plasma of T-ALL patients, highlighting that lncRNAs may represent a new class of promising accurate and sensitive biomarkers in these young patients. Thus, the unveiling of the aberrant signature of circulating and intracellular levels of lncRNAs could have great clinical utility for obtaining a more accurate definition of prognosis and uncovering novel therapeutic strategies against T-ALL in children. However, further investigations are needed to better define the standard methodological procedure for their quantification and to obtain their specific targeting in T-ALL pediatric patients.

## 1. Introduction

T acute lymphoblastic leukemia (T-ALL) represents a rare variant in pediatric patients, with a frequency of approximately 15% of total cases of acute lymphoblastic leukemia. It presents an unusual morphology, with typical genetic and clinical characteristics that distinguish this subgroup from other non-T-cell hematological malignancies. T-ALL children are more frequently male, presenting leukocytosis and a mean age of 9 years old. 

Despite advances in diagnosis and treatment, in a small percentage of cases, the disease involves the central nervous system (CNS), which is mostly involved in relapses to pharmacological treatment based on chemotherapy [1,2,3]. 

Non-coding RNAs (ncRNAs) represent a heterogeneous class of RNA molecules that do not encode proteins but, through different mechanisms, modulate gene expression.

Beyond the tRNAs (~80 nucleotides, nt) and rRNAs (~1500 nt) involved in protein synthesis, the ncRNA family can be divided into two other categories based on different sizes and functions: small ncRNAs (shorter than 200 nt) and long ncRNAs (longer than 300 nt).

The small ncRNA group includes snoRNA (~ 90 nt), piRNA (~30 nt) and miRNA (~10–22 nt), while the long ncRNA group includes lncRNA (>200 nt), pseudogenes and circular RNA [4]. snoRNAs are molecules responsible for chemical modifications (as methylation or pseudouridylation) to ribosomal RNAs and transfer RNA targets, while piRNAs, binding to Piwi proteins, promote the silencing of gene expression [4].

In light of the increasing evidence regarding the potential utility of miRNAs and lncRNAs in clinical practice in the oncology field [5,6,7,8,9,10,11,12], here, we focus our research on the role of these specific ncRNAs in the biology of pediatric T-ALL. 

Through the progression of throughput sequencing, more than 1000 miRNAs have been detected in humans. These small molecules are single-stranded RNAs that, by targeting the 3′ untranslated (3′UTR) region of specific mRNA targets, negatively regulate their expression by degradation or by translational repression [13]. 

By contrast, lncRNAs can modulate gene expression through multiple mechanisms in which they interact with chromatin, transcription factors, RNA molecules and other regulatory proteins [14,15]. First, they may act as molecular sponges, sequestering miRNAs and preventing them from interacting with their mRNA targets, or they can function as precursors of miRNAs. Additionally, lncRNAs can bind transcription factors or other proteins, serving as decoys and sequestering these factors from chromatin. Lastly, lncRNAs can function as scaffolds, promoting the assembly of chromatin-remodeling complexes or directing transcription factors to specific genomic loci to regulate gene expression. 

Moreover, several studies have highlighted the pivotal role of these molecules in regulating hematopoietic and lymphoid differentiation, as well as the critical role of cell proliferation and apoptosis in the development and function of the immune system and blood cells. Beyond their involvement in normal cellular functions, deregulation of miRNAs and lncRNAs is detected in the plasma of pediatric T-cell leukemia and in cancer cells, where they act as tumor suppressors or oncogenes regulating a wide variety of processes [4,16,17,18]. Additionally, recent evidence also showed that the high heterogeneity of the clinicopathologic characteristics of T-ALL patients is modulated by lncRNAs, confirming their potential utility as novel markers and targetable molecules for this disease. 

In the context of childhood T-ALL, increasing evidence has revealed the role of these molecules in modulating tumorigenesis and metastasis through several ways, including the acquisition of drug resistance. Indeed, despite significant advances in treatment strategies, drug resistance remains a major challenge in achieving durable remissions and preventing relapse. Different mechanisms modulated by miRNAs and lncRNAs can regulate drug resistance and, specifically, chemoresistance, conferring a selective advantage to the neoplastic cell clone that allows the disease to progress [16].

Consequently, miRNAs and, most recently, lncRNAs are being actively investigated as both diagnostic biomarkers and therapeutic targets to improve risk stratification during diagnosis and to provide more appropriate treatment for these young patients. 

In light of this, we performed a comprehensive literature search interrogating the PubMed database using the following combination of terms: “miRNA (or microRNA) and pediatric T leukemia (or T-ALL)”, “ncRNA (or non-coding RNA) and pediatric T leukemia (or T-ALL)”, “lncRNA (or long non-coding RNA) and pediatric T leukemia (or T-ALL)”. 

Following this preliminary search, we excluded retracted articles and studies that did not specifically address pediatric leukemia or its subtypes. Afterward, we focused on pathogenic mechanisms, investigating in vitro and in vivo human biological samples of pediatric T-ALL patients.

In this review, we describe the possible role of ncRNAs in pediatric T-ALL by illustrating a potential NGS workflow to study these molecules in the clinical context of T-ALL. We then provide an overview of the current knowledge regarding lncRNAs and miRNAs in pediatric T-ALL patients, with a particular focus on their aberrant expression and on the molecular processes they influence. Finally, we delve into ncRNAs as a potential diagnostic tool in the clinical context of pediatric T-ALL patients.

## 2. Analytic Workflow to Enlighten the Roles of ncRNA in T-ALL

**Figure 1:**
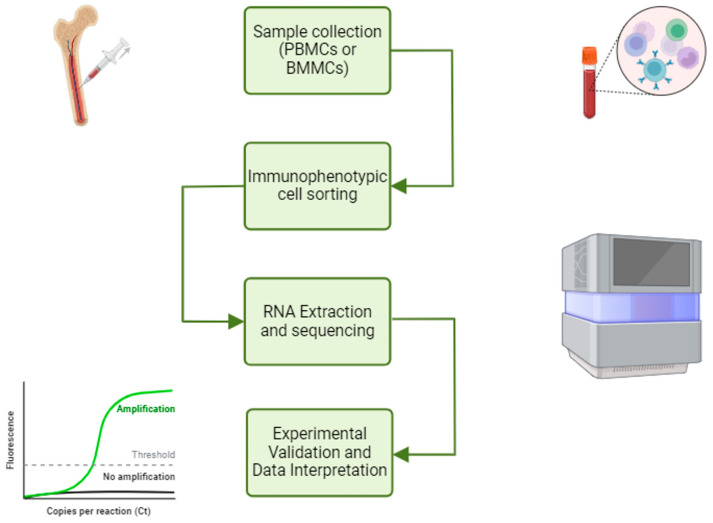
NGS workflow and ncRNA study. The NGS workflow for ncRNA study involves immunophenotyping cell sorting, RNA extraction and sequencing, differential expression analysis and experimental validation. This figure was created with BioRender (https://www.biorender.com, accessed on 15 March 2025).

A workflow strategy for the study of miRNAs and lncRNAs in T-ALL is shown in Figure 1. 

One aspect to consider regarding the diagnosis of T-ALL in pediatric patients is associated with the intrinsic heterogeneity of the disease. Indeed, in a biological sample of mononuclear cells obtained from bone marrow (BMMC) or peripheral blood (PBMC), not all mononuclear cells are leukemic blasts and, therefore, may present genetic mutations; on the contrary, other cells remain phenotypically normal. 

Therefore, RNA extraction from mononuclear cells can be inadequate for studying the differential expression analysis of disease-associated miRNAs and lncRNAs. 

The use of a cell sorter represents an advanced methodological step toward improving sample purity based on lineage-specific antigenic markers, as well as the use of a viability dye (i.e., 7-aminoactinomycin D, exclusion for live-cell selection). 

After the sorting, the tumor cells can be subjected to RNA sequencing (RNA-seq) and compared with the non-pathological counterpart if present in the same sample type. This approach has the potential to mitigate some biases in differential expression analysis that arise due to variations in the proportion of tumor cells within samples. Without cell sorting, the increased expression of one gene product in one biological sample compared to another may simply reflect the higher proportion of tumor cells rather than a true biological difference. 

Commonly used PCR-based NGS protocols may face limitations when detecting miRNAs and lncRNAs. 

In particular, lncRNAs often contain repetitive regions, which may prevent proper amplification during PCR. Furthermore, these repetitive sequences may hinder the accurate alignment of sequencing reads to the reference genome, leading to the incomplete or incorrect detection of lncRNAs. 

Furthermore, short-read-based NGS methods may fail to identify certain types of lncRNA variants, such as deletions or complex structural rearrangements, because they are difficult to infer from short sequence fragments. 

Alternative technologies may be employed to overcome these challenges. Optical genome mapping (OGM) [19], for example, can identify larger structural variations when probes are specifically designed to target regions of lncRNAs. 

This method is particularly effective in highlighting deletions, duplications or rearrangements involving lncRNAs. On the other hand, long-read sequencing technologies offer the advantage of capturing both structural variants and single-nucleotide changes.

By generating longer reads, these methods can span repetitive or complex regions, facilitating the accurate detection of lncRNAs and their variants. However, a potential limitation of long-read sequencing is its high requirement for RNA input, which may limit its application in samples with limited RNA availability.

By combining these approaches, researchers can obtain a more comprehensive analysis of miRNAs and lncRNAs, addressing the inherent limitations of each method and ensuring the accurate characterization of both expression and structural changes.

Regarding the differential expression analysis conducted on leukemia cell lines, the data obtained can present significant limitations due to the potential presence of mutations in genes that regulate the cell cycle and other key differentiation pathways. These mutations can cause aberrant expression profiles, including the overexpression of specific mRNAs, miRNAs or lncRNAs, which may not accurately reflect the physiological or pathological state observed in primary cells in tissues or biological fluids.

Consequently, such expression patterns can bias the analysis, leading to conclusions that are specific to the genetic background of the cell line rather than generalizable to the biological context under investigation. This highlights the importance of considering intrinsic genomic alterations in cell lines when interpreting differential expression results and, where possible, validating the results in more biologically relevant models. 

Therefore, depending on the availability of the biological sample and the specific scientific objectives of the study, different next-generation sequencing (NGS) techniques can be employed. Experimental validation is essential for validating the results of big data analyses, ensuring that the computationally derived insights are accurate and biologically relevant. This step is crucial for translating high-throughput data into meaningful conclusions that can lead to further scientific advances.

## 3. ncRNAs as Potential Therapeutic Target in Pediatric T-ALL Patients

**Figure 2:**
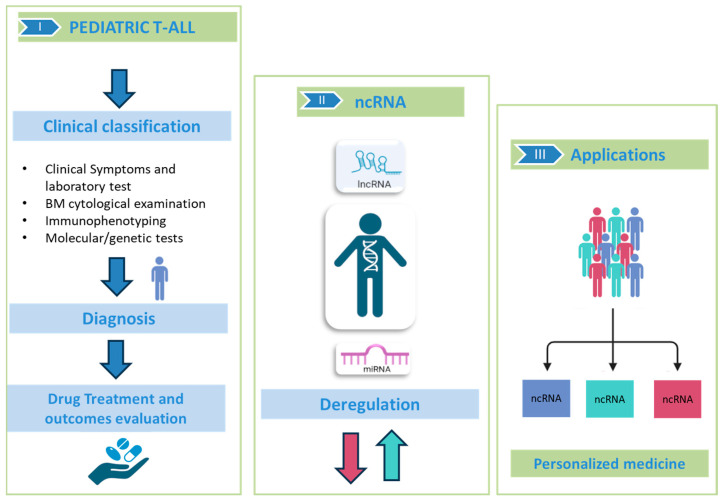
ncRNAs in pediatric T-ALL. In the specific sections: (I) Description of the gold standard for classification, diagnosis and prognosis of pediatric T-ALL through cytology and genetic tests; (II) Deregulation of ncRNA (as miRNAs and lncRNAs) involved in neoplastic progression; (III) ncRNAs may be useful for stratifying patients and promoting personalized medicine. This figure was created with BioRender https://www.biorender.com, “accessed on 15 March 2025”.

Although they do not encode proteins, miRNAs and lncRNAs can modulate various cellular functions that drive the malignant transformation of T-cell progenitors. The identification and study of deregulated ncRNAs could not only allow us to understand their role but also could allow for better patient stratification and the identification of alternative therapeutic strategies (Figure 2). 

Thus, based on their target genes, miRNAs and lncRNAs can be classified as oncogenes or tumor suppressors with respect to their contribution to the regulation of the hallmarks of cancer cells and, consequently, as emerging novel therapeutic targets for pediatric T-ALL patients.

### 3.1. Tumor-Suppressor ncRNAs

Loss in the expression of ncRNAs plays a critical and multifaceted role in the pathogenesis of T-ALL. In this context, by analyzing articles exclusively focused on childhood T-ALL patients, we found that several miRNAs act as tumor suppressors regulating the expression of several oncogenes involved in the leukemia progression (Table 1). 

Among tumor-suppressor miRNAs, miR-143-3p directly regulates the expression of KRAS, FGF1 and FGF9, inhibiting cell proliferation and viability [20].

Moreover, in silico analysis reveals the downregulation of miR-193-3p in pediatric T-ALL patients, while in vitro and in vivo experiments show that this miRNA reduced the onset of leukemia by targeting MYB [21]. The tumor-suppressor activity of this miRNA is confirmed, recently, by another study in which the downregulation of miR-193b-3p, induced by circ_0000745, increases the cell proliferation of T-ALL cells by releasing suppression of the known oncogene *NOTCH1* [22]. 

Finally, the loss of miR-203 expression inhibits apoptosis, which suggests its tumor-suppressor role in childhood T-ALL patients. Interestingly, the upregulation of miR-203 could be obtained by treating cells with COTI-2, a third-generation thiocarbazone that suppresses the growth of many cancers, including T-ALL cell lines, by activating p53 [23].

Interestingly, our search unveiled that tumor-suppressor activity in pediatric T-ALL patients is exerted mainly by miRNAs, while little or nothing is known regarding the tumor-suppressor activity of lncRNAs, as the studies that have emerged have unveiled their pivotal role mainly as oncogenes.

### 3.2. Oncogenic ncRNAs

Among the miRNAs deregulated in T-ALL patients, most of them exert an oncogenic activity (Table 2).

One of the dysregulated miRNAs in pediatric T-ALL is miR-125b, whose expression is significantly increased in T-leukemia homeobox 3 (TLX3)-positive T-ALL. TLX3, a transcription factor involved in T-cell development, by activating the lncRNA LINC00478, increases miR-125b expression. In vitro experiments and xenograft mouse models demonstrate that this miRNA targets the transcription factors Ets1 and CBFb, blocking normal T-cell differentiation and leading to an accumulation of immature T-cell progenitors [24].

Three independent studies suggested that miR-223-3p is a key regulator of pediatric T-ALL progression. Indeed, both Hou et al. [25] and Mansour et al. [26] showed that the high level of miR-223-3p downregulates the expression of FBW7, a key tumor suppressor in this cancer. Moreover, the overexpression of this miRNA is induced by the downregulation of circ_0000094 [25] or by the overexpression of the transcriptional factor TAL1 [26]. In the paper by Shu et al., in vivo and in vitro experiments show that miR-223-3p downregulated the tumor suppressor β-Arrestin1 (ARRB1), inhibiting leukemia progression [27].

Drobna et al. show that miR-20b-5p and miR-363-3p act simultaneously to reduce the expression of two well-known tumor suppressors, PTEN and BIM, promoting proliferation and inhibiting apoptosis [28]. A more recent work by Drobna reveals that the oncogenic role of this miRNA is mediated by the downregulation of PTPRC and SOCS2, which are involved in the inhibition of the JAK–STAT pathway [29].

Furthermore, the overexpression of miR-663b enhances proliferation and motility by regulating the expression of CD99, an important transmembrane protein involved in the aggressiveness of leukemia cells [30].

Finally, the metabolic regulation of leukemia cells is influenced by specific miRNAs. In pediatric T-ALL, the downregulation of the oncogenic miR-652-5p reduces glucose metabolism in T-ALL cells by targeting TIGAR, slowing the growth of leukemia cells in vivo and in vitro [31].

Also, lncRNAs are overexpressed in leukemia, where they can be categorized into different functional groups based on their role in (i) cell cycle alteration, (ii) the modulation of apoptosis and iii) drug resistance (Table 3).

Several key lncRNAs are overexpressed in T-ALL, influencing cell cycle regulation and contributing to the malignant phenotype in pediatric T-ALL. 

The lncRNA NALT instead promotes uncontrolled cell proliferation by regulating the NOTCH signaling pathway [32]. The ability of NALT to increase tumor growth was also confirmed in xenograft mice models [32].

The silencing of lncRNA T-ALL-R-LncR1, overexpressed in 11 cases out of 21 T-ALL children, induced apoptosis in Jurkat cells through the regulation of the pro-apoptotic protein PAR-4 [33].

On the other hand, other lncRNAs function as oncogenes, contributing to the resistance of leukemic cells to therapeutic interventions. In vitro and in vivo experiments reveal that the overexpression of lncRNA CDKN2B-AS1 is correlated with the resistance to adriamycin (ADR) by sponging miR-335-3p and inducing the expression of *TRAF5* [34].

## 4. ncRNAs as Potential Diagnostic Tool in Pediatric T-ALL Patients

It is well established that the expression profiles of miRNAs and lncRNAs are often specific and stable in blood and other biological fluids, where they can be detected and quantified using several non-invasive methods. 

For these characteristics, ncRNA have potential utility not only as therapeutic targets but also as potential biomarkers, offering an option for a potentially cost-effective approach to diagnosis and follow-up. While the identification of aberrant miRNAs in bone marrow requires an invasive analysis, the detection of circulating miRNAs in the plasma obtained from peripheral blood avoids an invasive procedure and allows for the unveiling of novel non-invasive biomarkers for early diagnosis and, in addition, the evaluation of leukemia relapse during the long-term follow-up of these young patients.

Indeed, the development of RNA sequencing (RNA-seq) technologies has allowed researchers to uncover miRNAs (Table 4) and lncRNAs (Table 5) altered in the plasma and in the bone marrow of pediatric T-ALL patients.

miR-363-3p [29], miR-663b [30] and miR-652-5p [31], previously mentioned for their oncogenic activity in T-ALL cells (Table 3), unveil their potential utility as candidate biomarkers. In detail, miR-363-3p is upregulated in 81 pediatric ALL vs. 17 healthy children deposited in the GEO dataset (GSE23024) [35]. These data are also confirmed by Drobna et al., who found miR-363-3p in the bone marrow of 34 pediatric T-ALL patients vs. five healthy pediatric donors [28]. Still, miR-663b [30] and miR-652-5p are upregulated in the blood of 30 and 13 pediatric T-ALL patients, respectively. 

miR-196b is overexpressed in leukemic cells extracted from the bone marrow of 22 pediatric T-ALL patients compared to 50 precursor B-ALL cells, and this overexpression is correlated with the aberrant activation of HOXA genes [36].

Finally, in a comparison of miRNA profiles between T-ALL patients and healthy children, miR-652-5p upregulation emerged in children affected by T-ALL [31]. 

A recent observational study reported that the polymorphism of MEG3 (rs7158663 AG/AA) is correlated with higher susceptibility to ALL insurgence in childhood [37]. Consistent with the expression of H19 in adult ALL patients [42], this lncRNA is also detected as upregulated in pediatric patients, where it is negatively correlated with miR-326 and directly with BCL2, a known anti-apoptotic protein involved in the progression of this disease [38].

Regarding T-ALL subtypes, the previously mentioned lncRNAs (i.e., NALT [32], CDKN2B-AS1 [34]), beyond their oncogenic activity, have shown a potential employment as biomarkers, as their expression is upregulated in pediatric T-ALL patients compared to healthy controls. Moreover, CDKN2B-AS1 was found to be mainly upregulated in pediatric T-ALL patients who have acquired resistance to ADR, which suggests its contribution to drug resistance [34]. 

Also, the lncRNAs T-ALL-R-LncR1 [33] and LUNAR1 [39] were found to be overexpressed in childhood T-ALL. In particular, lncRNA T-ALL-R-LncR1 is overexpressed in 11 out of 21 pediatric T-ALL patients [33], while the high expression of LUNAR1 is positively correlated with NOTCH1 and IGF-1R and with poor prognosis, which suggests its potential role as a prognostic biomarker [39]. 

Comparing childhood T-ALL with B-ALL patients, specific lncRNAs have been found to have clinical relevance because they are differentially expressed between these two groups. In this context, in a recent paper by Sharm et al., RNA sequencing revealed the high expression of LINC01221, RP11-472G21.2 and CRNDE in pediatric T-ALL patients compared to B-ALL and to healthy controls [40]. Also, the higher expression of lncRNA uc.112, mapping on transcribed ultraconserved regions (T-UCRs), occurs in pediatric T-ALL compared to B-ALL patients [41].

### ncRNAs and Central Nervous System (CNS) Invasion in the Management of Pediatric T-ALL

Predicting and managing CNS recurrence remains one of the main challenges to increasing survival in T-ALL. Leukemic cells can avoid systemic therapy by infiltrating and persisting within the CNS. The lack of sensitive biomarkers makes it difficult to reliably identify children at risk of CNS relapse [43]. For this purpose, there are promising approaches involving novel drugs and flow cytometry analysis of cerebrospinal fluid, including ncRNA detection. ncRNAs influence the ability of leukemic cells to move and infiltrate the CNS, as well as to control their survival and proliferation. 

For example, the higher expression of miR-181a was found in pediatric acute lymphoblastic leukemia patients with CSN involvement, which suggests its potential value as a marker in liquid biopsy [44]. In addition, the downregulation of miR-34a promotes cell movement and facilitates the infiltration of leukemic cells into organs, including the CNS [45]. Other studies are underway to identify specific lncRNA signatures associated with a specific leukemia subtype and with CSN invasion in pediatric T-ALL [40,46]. 

In this scenario, future prospective studies could include the evaluation of ncRNAs to better identify children with acute lymphoblastic leukemia (ALL) who are at risk of CNS involvement.

## 5. Conclusions

MicroRNAs (miRNAs) are extensively studied in pediatric T acute lymphoblastic leukemia (T-ALL) due to their crucial regulatory roles in various biological processes. Evidence links specific miRNAs to poor prognosis and therapeutic resistance, underscoring their potential as biomarkers and therapeutic targets. Similarly, long non-coding RNAs (lncRNAs) have emerged as key regulators in leukemia biology. Distinct lncRNA expression signatures across T-ALL subtypes provide valuable prognostic insights, which reinforces their promise as diagnostic and therapeutic tools.

Despite encouraging preclinical findings, a critical translational gap remains. This report presents a comprehensive review of the scientific literature and highlights the absence of clinical trials assessing the efficacy and safety of ncRNA-targeted therapies or monitoring strategies in pediatric T-ALL. This lack of clinical validation hinders the integration of lncRNA-based approaches into standardized diagnostic and therapeutic protocols. To address this gap, rigorous preclinical investigations are essential for establishing the safety, efficacy and long-term impact of lncRNA-based interventions. Additionally, developing standardized protocols for lncRNA diagnostics and therapeutics is crucial for advancing personalized treatment strategies in pediatric T-ALL.

Furthermore, our analysis of the current literature indicates that miRNAs often exhibit tumor-suppressive activity, whereas many lncRNAs are primarily associated with leukemogenesis, disease progression and poor outcomes. Therefore, additional investigation is needed to explore the crosstalk between miRNAs and lncRNAs in sustaining T-ALL cell growth and progression, as well as their potential as biomarkers for predicting therapeutic responses, making them valuable diagnostic and prognostic markers in pediatric T-ALL.

In conclusion, we hope this review, focusing on the molecular mechanisms of miRNAs and lncRNAs, will inspire innovative approaches to developing more effective treatment strategies and diagnostic tools, ultimately improving clinical outcomes for pediatric T-ALL patients.

## Figures and Tables

**Table 1:** Tumor-suppressor miRNAs in pediatric T-ALL patients.

miRNAs	Regulator	Target Genes	Function	Experimental Model	Ref.
miR-143-3p	ns	*KRAS*, *FGF1* and *FGF9*	Inhibits T-ALL proliferation	In vitro	[20]
miR-193b-3p	ns	*MYB*	Inhibits T-ALL onset	In vitro and in vivo	[21]
	circ_0000745	*NOTCH1*	Inhibits T-ALL proliferation	In vitro	[22]
miR-203	COTI-2 treatment	ns	Promotes apoptosis	In vitro and in vivo	[23]

ns: not specified.

**Table 2:** OncomiRs in pediatric T-ALL patients.

miRNAs	Regulator	Target Genes	Function	Experimental Model	Ref.
miR-125b	TLX3/ LINC00478	*Ets1*, *CBFb*	Impairs T-cell differentiation	In vitro and in vivo	[24]
miR-223-3p	circ_0000094	*FBW7*	Promotes T-ALL proliferation, migration and invasion, reduces apoptosis	In vitro	[25]
	TAL1	*FBW7*	Promotes T-ALL proliferation, reduces apoptosis	In vitro	[26]
	ns	*ARRB1*	Promotes T-ALL progression	In vitro and in vivo	[27]
miR-20b-5p/ miR-363-3p	ns	*PTEN*, *BIM*	Enhance T-ALL proliferation, inhibit apoptosis	In vitro	[28]
miR-363-3p	Promoter hypermethylation	*PTPRC* and *SOCS2*	Enhances cell growth by inhibiting JAK–STAT pathway	In vitro	[29]
miR-663b	ns	*CD99*	Enhances proliferation and motility, inhibits apoptosis	In vitro	[30]
miR-652-5p	ns	*TIGAR*	Reduces glucose metabolism, slowing leukemia progression	In vitro and in vivo	[31]

ns: not specified.

**Table 3:** Oncogenic lncRNAs in pediatric T-ALL patients.

lncRNAs	Regulator	Target Genes	Function	Experimental Model	Ref.
NALT	ns	*NOTCH1*	Cell proliferation	In vivo and in vitro	[32]
T-ALL-R-LncR1	ns	*PAR-4*	Apoptosis	In vitro	[33]
CDKN2B-AS1	ns	miR-335-3p	Drug resistance	In vivo and in vitro	[34]

ns: not specified.

**Table 4:** miRNAs as potential candidate biomarkers in pediatric T-ALL patients.

miRNAs	Expression	Pediatric Cohort	Methods	Ref.
miR-363-3p	Up	34 T-ALL vs. 5 healthy children	q-RT-PCR	[28]
		81 ALL vs. 17 healthy children	microRNA array	[35]
miR-663b	Up	30 T-ALL vs. 30 healthy donors	q-RT-PCR	[30]
miR-196b	Up	22 T-ALL vs. 50 B-ALL	q-RT-PCR	[36]
miR-652-5p	Up	13 T-ALL vs. 5 healthy children	q-RT-PCR	[31]

**Table 5:** lncRNAs as potential biomarker in pediatric T-ALL patients.

lncRNAs	Expression	Pediatric Cohort	Methods	Ref.
MEG3 rs7158663 AG/AA	Higher susceptibility	266 ALL vs. matched healthy controls	q-RT-PCR	[37]
H19	Up	37 ALL vs. 17 healthy controls	In silico and q-RT-PCR	[38]
NALT	Up	20 T-ALL vs. 10 healthy controls	q-RT-PCR	[32]
CDKN2B-AS1	Up	42 T-ALL vs.20 healthy controls 21 T-ALL ADR resistance vs. 21 T-ALL non-ADR	q-RT-PCR	[34]
T-ALL-R-LncR1	Up	21 T-ALL	q-RT-PCR	[33]
LUNAR1	Up	185 T-ALL vs. 40 healthy controls	q-RT-PCR	[39]
LINC01221, RP11-472G21.2 CRNDE	Up	51 T-ALL vs. 32 B-ALL vs.16 healthy controls	RNA sequencing	[40]
lncRNA uc.112,	Up	32 T-ALL vs.30 B-ALL	q-RT-PCR	[41]

ADR: adriamycin.

## Data Availability

Data derived from public domain resources.

## Abbreviations

The following abbreviations are used in this manuscript:

ADR	Adriamycin
BMMC	Mononuclear cells from bone marrow
CNS	Central nervous system
LncRNA	Long non-coding RNA
miRNA	microRNA
ncRNA	Non-coding RNA
NGS	Next-generation sequencing
OGM	Optical genome mapping
PBMC	Mononuclear cells from peripheral blood
RNA-seq	RNA-sequencing
T-ALL	T acute lymphoblastic leukemia

## References

[1] Lato M.W., Przysucha A., Grosman S., Zawitkowska J., Lejman M. (2021). The New Therapeutic Strategies in Pediatric T-Cell Acute Lymphoblastic Leukemia. Int. J. Mol. Sci..

[2] Ekpa Q.L., Akahara P.C., Anderson A.M., Adekoya O.O., Ajayi O.O., Alabi P.O., Okobi O.E., Jaiyeola O., Ekanem M.S. (2023). A Review of Acute Lymphocytic Leukemia (ALL) in the Pediatric Population: Evaluating Current Trends and Changes in Guidelines in the Past Decade. Cureus.

[3] Inaba H., Pui C.H. (2021). Advances in the Diagnosis and Treatment of Pediatric Acute Lymphoblastic Leukemia. J. Clin. Med..

[4] Slack F.J., Chinnaiyan A.M. (2019). The Role of Non-coding RNAs in Oncology. Cell.

[5] Toden S., Zumwalt T.J., Goel A. (2021). Non-coding RNAs and potential therapeutic targeting in cancer. Biochim. Biophys. Acta Rev. Cancer.

[6] Dakal T.C., Philip R.R., Bhushan R., Sonar P.V., Rajagopal S., Kumar A. (2025). Genetic and epigenetic regulation of non-coding RNAs: Implications in cancer metastasis, stemness and drug resistance. Pathol. Res. Pract..

[7] Ghazimoradi M.H., Karimpour-Fard N., Babashah S. (2023). The Promising Role of Non-Coding RNAs as Biomarkers and Therapeutic Targets for Leukemia. Genes.

[8] Orlandella F.M., Mariniello R.M., Mirabelli P., De Stefano A.E., Iervolino P.L.C., Lasorsa V.A., Capasso M., Giannatiempo R., Rongo M., Incoronato M. (2020). miR-622 is a novel potential biomarker of breast carcinoma and impairs motility of breast cancer cells through targeting NUAK1 kinase. Br. J. Cancer.

[9] Orlandella F.M., Imperlini E., Pane K., Luciano N., Braile M., De Stefano A.E., Iervolino P.L.C., Ruocco A., Orrù S., Franzese M. (2024). miR-331-5p Affects Motility of Thyroid Cancer Cell Lines and Regulates BID Expression. Biomedicines.

[10] Braile M., Luciano N., Carlomagno D., Salvatore G., Orlandella F.M. (2024). Insight into the Role of the miR-584 Family in Human Cancers. Int. J. Mol. Sci..

[11] Hussen B.M., Hidayat H.J., Salihi A., Sabir D.K., Taheri M., Ghafouri-Fard S. (2021). MicroRNA: A signature for cancer progression. Biomed. Pharmacother..

[12] Rupaimoole R., Slack F.J. (2017). MicroRNA therapeutics: Towards a new era for the management of cancer and other diseases. Nat. Rev. Drug Discov..

[13] Saliminejad K., Khorram Khorshid H.R., Soleymani Fard S., Ghaffari S.H. (2019). An overview of microRNAs: Biology, functions, therapeutics, and analysis methods. J. Cell Physiol..

[14] Wang K.C., Chang H.Y. (2011). Molecular mechanisms of long noncoding RNAs. Mol. Cell.

[15] Hu G., Niu F., Humburg B.A., Liao K., Bendi S., Callen S., Fox H.S., Buch S. (2018). Molecular mechanisms of long noncoding RNAs and their role in disease pathogenesis. Oncotarget.

[16] Sharma S. (2024). Unraveling the role of long non-coding RNAs in therapeutic resistance in acute myeloid leukemia: New prospects & challenges. Noncoding RNA Res..

[17] Orlandella F.M., Smaldone G., Salvatore G., Vitagliano L., Cianflone A., Parasole R., Beneduce G., Menna G., Salvatore M., Mirabelli P. (2021). The lncRNA TEX41 is upregulated in pediatric B-Cells Acute Lymphoblastic Leukemia and it is necessary for leukemic cell growth. Biomark Res..

[18] Affinito O., Pane K., Smaldone G., Orlandella F.M., Mirabelli P., Beneduce G., Parasole R., Ripaldi M., Salvatore M., Franzese M. (2020). lncRNAs-mRNAs Co-Expression Network Underlying Childhood B-Cell Acute Lymphoblastic Leukaemia: A Pilot Study. Cancers.

[19] Dremsek P., Schwarz T., Weil B., Malashka A., Laccone F., Neesen J. (2021). Optical Genome Mapping in Routine Human Genetic Diagnostics-Its Advantages and Limitations. Genes.

[20] Dawidowska M., Maćkowska-Maślak N., Drobna-Śledzińska M., Kosmalska M., Jaksik R., Szymczak D., Jarmuż-Szymczak M., Sadowska-Klasa A., Wojtaszewska M., Sędek Ł. (2022). Small RNA-Seq Reveals Similar miRNA Transcriptome in Children and Young Adults with T-ALL and Indicates miR-143-3p as Novel Candidate Tumor Suppressor in This Leukemia. Int. J. Mol. Sci..

[21] Mets E., Van der Meulen J., Van Peer G., Boice M., Mestdagh P., Van de Walle I., Lammens T., Goossens S., De Moerloose B., Benoit Y. (2015). MicroRNA-193b-3p acts as a tumor suppressor by targeting the MYB oncogene in T-cell acute lymphoblastic leukemia. Leukemia.

[22] Feng H., Li F., Tang P. (2021). Circ_0000745 regulates NOTCH1-mediated cell proliferation and apoptosis in pediatric T-cell acute lymphoblastic leukemia through adsorbing miR-193b-3p. Hematology.

[23] Guo Y., Zhu X., Sun X. (2020). COTI-2 induces cell apoptosis in pediatric acute lymphoblastic leukemia via upregulation of miR-203. Bioengineered.

[24] Renou L., Boelle P.Y., Deswarte C., Spicuglia S., Benyoucef A., Calvo J., Uzan B., Belhocine M., Cieslak A., Landman-Parker J. (2017). Homeobox protein TLX3 activates miR-125b expression to promote T-cell acute lymphoblastic leukemia. Blood Adv..

[25] Hou Y., Sun J., Huang J., Yao F., Chen X., Zhu B., Zhao D. (2021). Circular RNA circRNA_0000094 sponges microRNA-223-3p and up-regulate F-box and WD repeat domain containing 7 to restrain T cell acute lymphoblastic leukemia progression. Human Cell.

[26] Mansour M.R., Sanda T., Lawton L.N., Li X., Kreslavsky T., Novina C.D., Brand M., Gutierrez A., Kelliher M.A., Jamieson C.H. (2013). The TAL1 complex targets the FBXW7 tumor suppressor by activating miR-223 in human T cell acute lymphoblastic leukemia. J. Exp. Med..

[27] Shu Y., Wang Y., Lv W.Q., Peng D.Y., Li J., Zhang H., Jiang G.J., Yang B.J., Liu S., Zhang J. (2020). ARRB1-Promoted NOTCH1 Degradation Is Suppressed by OncomiR miR-223 in T-cell Acute Lymphoblastic Leukemia. Cancer Res..

[28] Drobna M., Szarzyńska B., Jaksik R., Sędek Ł., Kuchmiy A., Taghon T., Van Vlierberghe P., Szczepański T., Witt M., Dawidowska M. (2020). hsa-miR-20b-5p and hsa-miR-363-3p Affect Expression of PTEN and BIM Tumor Suppressor Genes and Modulate Survival of T-ALL Cells In Vitro. Cells.

[29] Drobna-Śledzińska M., Maćkowska-Maślak N., Jaksik R., Kosmalska M., Szarzyńska B., Lejman M., Sędek Ł., Szczepański T., Taghon T., Van Vlierberghe P. (2022). Multiomics to investigate the mechanisms contributing to repression of PTPRC and SOCS2 in pediatric T-ALL: Focus on miR-363-3p and promoter methylation. Genes Chromosomes Cancer.

[30] Liu X., Zhang H., Zhang B., Zhang X. (2019). Expression and Role of MicroRNA-663b in Childhood Acute Lymphocytic Leukemia and its Mechanism. Open Med..

[31] Liu S., Wang H., Guo W., Zhou X., Shu Y., Liu H., Yang L., Tang S., Su H., Liu Z. (2022). MiR-652-5p elevated glycolysis level by targeting TIGAR in T-cell acute lymphoblastic leukemia. Cell Death Dis..

[32] Wang Y., Wu P., Lin R., Rong L., Xue Y., Fang Y. (2015). LncRNA NALT interaction with NOTCH1 promoted cell proliferation in pediatric T cell acute lymphoblastic leukemia. Sci. Rep..

[33] Zhang L., Xu H.G., Lu C. (2014). A novel long non-coding RNA T-ALL-R-LncR1 knockdown and Par-4 cooperate to induce cellular apoptosis in T-cell acute lymphoblastic leukemia cells. Leuk. Lymphoma.

[34] Chen L., Shi Y., Li J., Yang X., Li R., Zhou X., Zhu L. (2020). LncRNA CDKN2B-AS1 contributes to tumorigenesis and chemoresistance in pediatric T-cell acute lymphoblastic leukemia through miR-335-3p/TRAF5 axis. Anticancer Drugs.

[35] Schotte D., e Menezes R.X., Akbari Moqadam F., Khankahdani L.M., Lange-Turenhout E., Chen C., Pieters R., Den Boer M.L. (2011). MicroRNA characterize genetic diversity and drug resistance in pediatric acute lymphoblastic leukemia. Haematologica.

[36] Schotte D., Lange-Turenhout E.A., Stumpel D.J., Stam R.W., Buijs-Gladdines J.G., Meijerink J.P., Pieters R., Den Boer M.L. (2010). Expression of miR-196b is not exclusively MLL-driven but is especially linked to activation of HOXA genes in pediatric acute lymphoblastic leukemia. Haematologica.

[37] Pei J.S., Chang W.S., Chen C.C., Mong M.C., Hsu S.W., Hsu P.C., Hsu Y.N., Wang Y.C., Tsai C.W., Bau D.T. (2022). Novel Contribution of Long Non-coding RNA MEG3 Genotype to Prediction of Childhood Leukemia Risk. Cancer Genom. Proteom..

[38] Mofidi M., Rahgozar S., Pouyanrad S. (2021). Increased level of long non coding RNA H19 is correlated with the downregulation of miR-326 and BCL-2 genes in pediatric acute lymphoblastic leukemia, a possible hallmark for leukemogenesis. Mol. Biol. Rep..

[39] El-Khazragy N., Abdel Aziz M.A., Hesham M., Matbouly S., Mostafa S.A., Bakkar A., Abouelnile M., Noufal Y., Mahran N.A., Abd Elkhalek M.A. (2021). Upregulation of leukemia-induced non-coding activator RNA (LUNAR1) predicts poor outcome in pediatric T-acute lymphoblastic leukemia. Immunobiology.

[40] Sharma PKaur P., Bhatia P., Trehan A., Sreedharanunni S., Singh M. (2024). Novel lncRNAs LINC01221, RP11-472G21.2 and CRNDE are markers of differential expression in pediatric patients with T cell acute lymphoblastic leukemia. Cancer Cell Int..

[41] das Chagas P.F., de Sousa G.R., Kodama M.H., de Biagi Junior C.A.O., Yunes J.A., Brandalise S.R., Calin G.A., Tone L.G., Scrideli C.A., de Oliveira J.C. (2021). Ultraconserved long non-coding RNA uc.112 is highly expressed in childhood T versus B-cell acute lymphoblastic leukemia. Hematol. Transfus. Cell Ther..

[42] Asadi M., Gholampour M.A., Kompani F., Alizadeh S. (2023). Expression of Long Non-Coding RNA H19 in Acute Lymphoblastic Leukemia. Cell J..

[43] Thastrup M., Duguid A., Mirian C., Schmiegelow K., Halsey C. (2022). Central nervous system involvement in childhood acute lymphoblastic leukemia: Challenges and solutions. Leukemia.

[44] Egyed B., Kutszegi N., Sági J.C., Gézsi A., Rzepiel A., Visnovitz T., Lőrincz P., Müller J., Zombori M., Szalai C. (2020). MicroRNA-181a as novel liquid biopsy marker of central nervous system involvement in pediatric acute lymphoblastic leukemia. J. Transl. Med..

[45] Li X.J., Ren Z.J., Tang J.H. (2014). MicroRNA-34a: A potential therapeutic target in human cancer. Cell Death Dis..

[46] Buono L., Iside C., De Matteo A., Stellato P., Beneduce G., de Vera d’Aragona R.P., Parasole R., Salvatore M., Smaldone G., Mirabelli P. (2022). Specific lncRNA signatures discriminate childhood acute leukaemias: A pilot study. Cancer Cell Int..

